# Optical Measurement of Ligament Strain: Opportunities and Limitations for Intraoperative Application

**DOI:** 10.3390/s23177487

**Published:** 2023-08-28

**Authors:** Christian Marx, Paul Wulff, Christian Fink, Daniel Baumgarten

**Affiliations:** 1 Research Unit for Orthopedic Sports Medicine and Injury Prevention, UMIT TIROL—Private University for Health Sciences and Health Technology, 6060 Hall in Tirol, Austria; christian.marx@umit-tirol.at (C.M.); c.fink@gelenkpunkt.com (C.F.); 2 Chair of Mechatronics and Machine Dynamics, Technische Universität Berlin, 10623 Berlin, Germany; 3 Institute of Electrical and Biomedical Engineering, UMIT TIROL—Private University for Health Sciences and Health Technology, 6060 Hall in Tirol, Austria

**Keywords:** tendon strain, confocal chromatic imaging, shearography, digital image correlation (DIC), non-contact strain measurement

## Abstract

A feasible and precise method to measure ligament strain during surgical interventions could significantly enhance the quality of ligament reconstructions. However, all existing scientific approaches to measure in vivo ligament strain possess at least one significant disadvantage, such as the impairment of the anatomical structure. Seeking a more advantageous method, this paper proposes defining medical and technical requirements for a non-destructive, optical measurement technique. Furthermore, we offer a comprehensive review of current optical endoscopic techniques which could potentially be suitable for in vivo ligament strain measurement, along with the most suitable optical measurement techniques. The most promising options are rated based on the defined explicit and implicit requirements. Three methods were identified as promising candidates for a precise optical measurement of the alteration of a ligaments strain: confocal chromatic imaging, shearography, and digital image correlation.

## 1. Introduction

Joint injuries are among the most prevalent health issues. In 2021, over 189,000 surgical interventions for arthroscopic reconstruction of ligaments in the shoulder and the knee joint were conducted in German hospitals. These procedures are among the twenty most common, as reported by the Federal Statistical Office of Germany [1]. 

Joint structures with several biomechanical degrees of freedom, like the human knee, are mechanically in a state of overdetermination of equilibrium, if they are motionless [2]. Numerous anatomical structures contribute to this balance, including osteogenic and muscular structures, as well as tendons, ligaments, and the capsule apparatus. When one or more of these structures are traumatically damaged due to acute or chronic overload, surgical intervention often becomes necessary. Restoring the initial equilibrium state in ligament reconstructions poses a significant challenge for surgeons. Specifically, it is exceedingly difficult to achieve a strain state in the reconstructed ligament that aligns with the intact, interacting structures without accurately determining the actual strain states.

To date, there is no technique for an interoperative strain measurement that is implemented in the clinical routine. All known methods possess at least one disadvantage such as irreversible application of measurement equipment, damage to ligament tissue, inadequate measurement accuracy (see Section 2.2), requiring high technical and economical efforts, or being too time-consuming for clinical routine. In our quest for a suitable measurement method, we have listed our mandatory objectives in Table 1. We propose a measurement of the strain of the ligaments surface and additionally a measurement of the alteration of the strain state to be sufficient.

**Figure 1:**
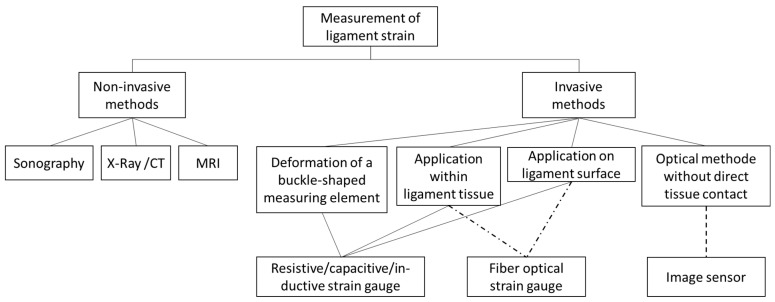
Classification of measurement techniques of ligament strain.

Figure 1 presents a classification of methods utilized for measuring ligament strain in both clinical routine and medical research. In clinical routine, non-invasive methods are employed to measure ligament strain. The use of magnetic resonance imaging (MRI) and computed tomography (CT) within a surgical intervention requires significant technical and financial resources. As such, these two techniques cannot be deemed suitable for interoperative strain measurements since they fail to meet objective No. 7 in Table 1. Sonography, although technically less complex with new techniques for tendon-motion tracking as seen in [3], achieves a high accuracy of up to 0.05 mm (mean average error). However, these do not meet the measurement accuracy requirements outlined in Section 2.2 for the planned application. Consequently, we exclude non-invasive methods from our screening.

Numerous invasive surgical procedures for measuring ligament strain have been developed in medical science [4,5,6,7,8,9,10,11,12,13,14,15,16,17,18,19,20,21,22,23,24,25,26,27,28,29,30,31,32,33,34]. The methods detailed in references [4,5,6,7] utilize resistive strain gauges made of mercury-filled silicon tubes, known as Liquid Metal Strain Gauges (LMSG), which were sutured directly to the tissue. In contrast, the Hall Effect Strain Transducers (HEST) described in [8,9,10,11,12] were affixed to ligaments or tendons using barbs. Although the setups in [11,12,13,14,15,16,17,18,19,20] employ the same attachment technique, they measure using Differential Variable Reluctance Transducers (DVRT). The studies in [21,22,23,24,25] used adhesively attached resistive strain gauges, crafted from silicon with a notably low Young’s modulus. Optical fibers, embedded with a Bragg grating and serving as strain sensors, were the choice in [26,27,28] and were adhesively bonded to the tissue. An interesting approach is seen in [29,30], where an optical isotropic polymeric coating is applied to the tissue. When this coating is illuminated with coherent light, the reflective optical signal conveys the strain information. Lastly, references [31,32,33,34] utilized digital image correlation (DIC), leveraging images captured via Charge-Coupled Device (CCD) or Complementary Metal-Oxide-Semiconductor (CMOS) sensors.

In all-known measurement methods possess at least one of the disadvantages listed in Table 2 and fail to meet the objectives defined in Table 1. Due to the damage to tissue resulting from the insertion of barbs, needles, suture material, or the application of irreversible adhesives, as well as considerable technical effort and time expenditure, these procedures are unsuitable for clinical routine.

An intraoperative strain measurement that is suitable for clinical routine would be a significant improvement for the aforementioned medical interventions. Ensuring a uniform strain state in paired anatomical structures, such as the collateral ligaments of the knee, is crucial for the healing process and postoperative joint stability. To date, no suitable measuring instruments or methods are available for clinical routine (see Table 2). The scientific methods utilized in [4,5,6,7,8,9,10,11,12,13,14,15,16,17,18,19,20,21,22,23,24,25,26,27,28,29,30,31,32,33,34] are time-consuming, the measurement results are influenced by the measurement setup, and the setup irreversibly damages the examined tissue.

Two methods, polymeric strain gauges and optical sensors, appear most promising. Given the advanced state of technical development of commercially available endoscopic camera systems, we focus on an optical measurement system in this publication. 

This article is structured as follows: In Section 2, technical requirements are defined, which serve as hard criteria for the screening of applicable technical systems conducted in Section 3. In Section 4, the screening results are assessed in terms of their applicability, leading to the conclusions presented in Section 5.

## 2. Determination of Technical Requirements

The prerequisites for a non-contact, optical measurement system for ligament strain, capable of measuring relative deviation, are outlined based on the proposed surgical workflow. The most stringent requirements are examined in detail to define both qualitative and quantitative criteria.

### 2.1. Postulated Workflow for an Intraoperative Strain Measurement of Ligaments and According Requirements

The following activities are postulated to form the essential workflow of an optical non-contact strain measurement of a ligament. Ideally those activities should be compatible with minimally invasive surgery (MIS) as this type of procedures is very common for ligament reconstructions. The accompanying requirements are listed in Table 3. Please note that, since an in vivo application is proposed, the temperature of the ligament surface is assumed to remain nearly constant throughout the measurement.

1. Sterilize surfaces of the surgical equipment.
2. Create surgical access.
3. Irrigation of measuring area.
4. First imaging of the measuring area.
5. Apply stoichiometric pattern on the relevant anatomical structure(s). *
6. Set the measuring area (manually).
7. First image measurement:
  (a) Optical measurement of the three-dimensional topology (3D-topology) (vertical measurement);
  (b) Optical measurement of positions of the stoichiometric pattern (lateral measurement).
8. First image analysis:
  (a) Analysis of the 3D-topology based on 7(a);
  (b) Assign the positions measured in 7(b) to the calculated surface of step 8(a).
9. Manual manipulation of the joint to alter the strain state of the ligament.
10. Second image measurement:
  (a) Optical measurement of the (altered) 3D-topology (vertical measurement);
  (b) Optical measurement of altered positions of the stoichiometric pattern (lateral measurement).
11. Second image analysis:
  (a) Analysis of the 3D-topology based on 10(a);
  (b) Assign the positions measured in 10(b) to the calculated surface of step 11(a);
  (c) Calculate the displacement vectors of the elements of the stoichiometric pattern based on 8(b) and 11(b);
  (d) Derive the alteration of the strain state of the measuring area via 11(c) in comparison to the measurement in step 8.
12. Diagramming the obtained data for the surgeon.

* Step five may not be required for some of the screened measurement techniques as they do not require an artificial stoichiometric pattern.

**Table 3:** Technical requirements for an optical strain measurement of ligaments.

No.	Description of Requirement	Referencing Step of Procedure
1	Sterilizability of the surfaces of invasive surgical equipment	Step 1
2	Highly compact design of invasive surgical equipment and small surgical access	Step 2
3	Compatibility of the measurement system with the optical properties of medical rinse (e.g., refractive index, adsorption, and speed of light within fluid)	Step 3
4	Biocompatibility/resorbability of the colorant of the stoichiometric pattern *	Step 5
5	Feasibility of surgical application of the stoichiometric pattern *	Step 5
6	Technical suitability of the stoichiometric pattern (e.g., high edge definition, contrast, and refractive index) *	Step 5
7	Graphical user interface (GUI): set measurement area	Step 6
8	Accuracy demand for measurement system	Step 7, 10
9	Nearly simultaneous imaging for step 7(a) and (b) as well as step 10(a) and (b) to avoid movement artifacts	Step 7, 10
10	Software requirements from: Step 4, 6, 7, 8(a,b) and 11(c,d)	Step 4, 6, 7, 8, 11
11	GUI: ascertainable evaluation of the measurement results	Step 12

* May not be required for some of the screened measurement techniques as they do not require an artificial stoichiometric pattern.

In identifying the most critical requirements, we assessed them as follows: The first three requirements are fundamental preconditions for surgical equipment used in MIS. Commercially available endoscopic systems meet these requirements [35]. Consequently, requirements 1–3 are not considered further as crucial for the defined task. Requirement 4 is also not considered limiting as biocompatible colorants for intraoperative use are available [36]. The application of a stoichiometric pattern (requirement 6) is not necessary for all types of optical measurement principles and does not seem to pose a constraint as biocompatible, sterile colorants are available [36]. Still, this requirement is technically challenging.

Requirements 7, 10, and 11 are common software prerequisites for optical measurement systems. State-of-the-art optical measurement systems, which are commercially available, meet these requirements [37]. Therefore, requirements 7, 10, and 11 are not viewed as pivotal. Requirement 8 is evaluated as crucial due to the small target area for measurements on smaller ligaments, such as the anterior cruciate ligament (ACL) of a human knee joint, which may not exceed an average spatial extent of 17.8 mm [38]. Moreover, a precise strain measurement for smaller strain rates is desirable for the defined application. The implicit requirement 9 is closely related to requirement 8 and serves as a benchmark for technical screening.

### 2.2. Requirements for Measurement Accuracy: Model Assumptions and Case Study

**Figure 2:**
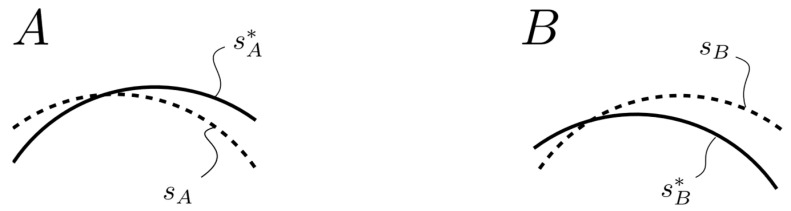
Configuration (**A**) (initial state) and (**B**) (altered state).

Measurement accuracy for this study is quantitatively benchmarked by using a model assumption for a strain measurement longitudinal to the fiber direction of the collagen tissue. Our objective is to perform precise strain measurement within the linear region of the stress–strain behavior of ligaments, as described in reference [39]. Accordingly, we model the material behavior as linearly elastic. Assumption I states that a strain measurement requires at least two images at different states of strain (A and B). A circular arc is proposed as the simplest model to describe a straight line on a curved surface. Hence, the ligament surface in our model is described via the arc length of a circular segment in a two-dimensional plane (Assumption II), as illustrated in Figure 2.

The calculated elongation is subject to measurement errors since it is based on the measurements described in procedure steps 7 and 10. So, here and in the following all actual quantities are marked with an asterisk whereas the measured quantities are not marked. The deviation of the strain state Δ*ε* is demonstrated below:

(1) $$\Delta \varepsilon =\left|\varepsilon -\varepsilon^{*}\right|.$$

The altered strain state of the examined ligament is gained via the altered arc length $s_{B}^{\left(*\right)}$ in comparison to the initial arc length $s_{A}^{\left(*\right)}$ after procedure step 9 (see Figure 2).

Accordingly, the altered strain state $\varepsilon^{\left(*\right)}$ of the ligament is calculated as

(2) $$\varepsilon^{\left(*\right)}=\frac{s_{B}^{\left(*\right)}-s_{A}^{\left(*\right)}}{s_{A}^{\left(*\right)}}\;.$$

The assumption is made that the measurement errors are all the same magnitude in the spatial coordinates (Assumption III):

(3) ∆x=∆y=∆z.

Please note that these measurement errors are not identical with the deformations of the measuring points. Based on assumption III, it is simplified that the resulting error ∆s of the arc length $s_{A}$ and $s_{B}$ are equal:

(4) $$∆s=\left|s_{A}^{*}-s_{A}\right|=\left|s_{B}^{*}-s_{B}\right|.$$

Based on Equation (2) plus a consideration of the extreme case, which can be found within the Appendix A, the following equation is gained:

(5) $$∆\varepsilon =\frac{∆s(2+\varepsilon )}{s_{A}^{*}-∆s}\;.$$

At least the spatial coordinates of three points (point 1, 2, and 3) on the circular segment are required to fully, mathematically describe the arc length $s_{A}$ or ${\;s}_{B}$. The related error calculation and the accompanying considerations are presented within the Appendix A. Based on the error calculation and Equation (3) we gain the final relationship of the deviation of the strain state Δε to the spatial error ∆x of the optical measurement system as expressed below:

(6) $$∆x=\frac{s_{A}^{*}∆\varepsilon }{5\left(2+∆\varepsilon +\varepsilon \right)}.$$

The maximum strain of ligaments varies significantly depending on the type of ligament. Reference [6] reports a maximum strain of up to 20% for lateral ankle ligaments. Meanwhile, significantly higher maximum strain values, up to 100%, were found in reference [5] for human anterior cruciate ligaments (ACL). Our application aims to be suitable for ligaments demonstrating lower strain values.

Two objectives are set: an ideal one and a minimum level of measurement accuracy for the intended application. Ideally, the measurement setup should be able to detect minor changes in the strain state with high accuracy. An alteration in the strain state $\varepsilon_{id}$ of 5% is expected to be measured with an accuracy of 2% regarding the measurement result of $\varepsilon_{id}$. This leads to an allowable deviation of ${∆\varepsilon }_{id}$ of 0.001. Additionally, this method was capable to measure within a small area. The posterior cruciate ligament is chosen as benchmark for the ideal case. Its average length according to [38] is 17.8 mm. As we cannot expect to utilize its whole length as a measurement area, an initial arc length $\;s_{A,\;id}^{*}$ of 10 mm is assumed. As a minimal goal an altered strain state $\varepsilon_{min}$ of 10 % is measured with an accuracy of 10 % at a larger initial arc length $s_{A,\;min}^{*}$ of 20 mm. Thus, the allowable deviation of ${∆\varepsilon }_{min}$ is 0.01. The inseted values and the calculated results of the maximum permissible error of the measuring system ∆x for both cases are depicted in Table 4.

### 2.3. Requirements for Image Resolution: Field-of-View

The image’s pixelation in the *xy*-plane should be equal to or smaller than the maximum permissible error. We utilize the calculated values for the maximum permissible error of the measuring system ∆x to estimate a minimum image pixelation for the lateral field of view (FoV). This measurement is carried out within the *xy*-plane, which is orthogonal to the *xz*-plane discussed above. The camera’s position remains unaltered for the two image measurements of procedure steps 7 and 10 as described in Section 2.1.

We anticipate a change in the measuring area’s position due to a shift in measuring points 1, 2, and 3 and a possible alteration in the *xz*-plane’s angle due to potential twisting of the ligament. An additional longitudinal spatial extension of the FoV, fifty percent more than the initial measuring length $s_{A}^{*}$, is estimated to be sufficient. To capture all measuring points within the second image measurement of procedure step 10, even if potential twisting of the ligament occurs, we estimate a transverse extension of the FoV to be at least one-third of the initial measuring length $s_{A}^{*}$.

The derived minimum image pixelation of the *xy*-plane is shown in Table 5. We calculate the pixelations by dividing the associated spatial extension by the maximum permissible error of the measuring system ∆x. The total pixelation is calculated by multiplying the two spatial extents in the *xy*-plane. The results are displayed to two decimal places.

## 3. Screening for Applicable Technical Systems

### 3.1. Screening Method for Medical Endoscopic Systems

Given that all methods described in Section 1 do not fulfill the requirements outlined in Section 2, a literature search was conducted via PubMed [40] on 3 June 2021. The aim was to identify medical endoscopic systems for both human and veterinary applications that, while not currently used for strain measurement, may potentially be applicable. This search included endoscopic systems not yet employed for in vivo imaging, as we sought systems with potential utility for our planned application. Accordingly, these systems would necessitate technical modifications. The search terms used are listed in Table 6.

### 3.2. Screening Method for Non-Medical Optical Measurement Techniques

Using the case study for maximum permissible error of the measuring system Δx from Section 2.2 as a reference, potentially suitable optical measurement systems were identified in [41]. The screening aimed to evaluate the feasibility of a measurement technique. All methods described would require several technical modifications to adapt to an endoscopic system. The source [41] is considered comprehensive in terms of optical measurement systems for technical and industrial applications. The inclusion criterion was a measurement accuracy that, at the very least, meets the required accuracy of the minimal scenario, as discussed in Section 2.2. A slight simplification was applied to the values of maximum permissible error of the measuring system Δx in the subsequent screening. The maximum permissible error for the ideal case was set to 0.001 mm and that for the minimal case to 0.02 mm (refer to Table 4). A quality distinction was made based on these two scenarios. As an exclusion criterion, the maximum detectable imaging area of the respective method was compared with the required FoVs of the described scenario. Here, the FoV of the ideal scenario forms the minimum requirement and that of the minimum scenario the ideal requirement. Each measurement technique that met these criteria was further examined in Section 4 for technical suitability within the planned application described in Section 2.1.

### 3.3. Screening Results of the Medical Endoscopic Systems

The results of the recherche according to Section 3.1 are listed in Table 7. 

### 3.4. Preselection of Non-Medical Measuring Techniques Based on Achievable Measurement Accuracy and Field-of-View

The screening results according to Section 3.2 taken from [41] are listed in Table 8. Please note, that the required FoV the minimal case is larger than the one of the ideal case (see Section 2.3). The optical measurements of strain measurement in [41] are rated according to their measurement accuracy of strain rates. Thus, all methods described for strain measurement are examined separately in Section 4.2. Additionally, to the methods described below, photoelasticity and the thermoelastic method are examined in detail.

## 4. Evaluation and Discussion 

### 4.1. Evaluation of the Medical Endoscopic Systems

The methods described in [45,47,48,49,50,55,56,58,59] do not meet the requirements for the measurement accuracy. Those described in [51,56,59,60] have an insufficient FoV for the planned application. The methods described in [43,45,51,52,53] are unsuitable for the analysis of displacement vectors. Nevertheless, the holographic endoscopic system described in [50] meets the minimal requirement regarding the measurement accuracy. The applicability of the holographic system is discussed in Section 4.2. None of the screened endoscopic systems are suitable for the planned application.

### 4.2. Evaluation of Non-Medical Measuring Techniques

All measurement techniques listed in Table 8 in Section 3.4 that fulfill at least the minimal requirement regarding the measurement accuracy and the minimal requirement regarding the FoV are examined further in this section and categized with respect to their suitability. Additionally, the optical measurement techniques of stain measurement described in [41] are scrutinized.

#### 4.2.1. Promising Candidates

When utilizing *confocal chromatic imaging*, the lateral displacement measurement is only possible using 3D markers. The technique fully meets the requirements for measurement accuracy. However, a limitation is that it is impossible to capture an image that encompasses the entire FoV of the minimal case, which is larger than that of the ideal case. Nonetheless, acquiring precise positional data within the FoV of the ideal case enables accurate strain calculations. Thus, the use of confocal chromatic sensors appears suitable in principle.

*Shearography* fully meets the criteria set for the screening. However, the following disadvantages posed technical challenges for the planned application: the required laser light is considered problematic as it may harm the tissue of the proband. A temporally synchronous surface acquisition is possible [41]. High strain rates can only be detected with continuous measurement [41]. Shearography appears suitable in principle, yet technically elaborate.

A *Digital image correlation (DIC)* via an endoscopic camera containing CMOS or CCD sensors does not yet offer the potential to fulfill the requirement of an ideal case. For image correlation the measurement accuracy is directly related to the resolution of the image sensors [41]. Accordingly, the considerations in Section 2.2 apply. The image sensor resolution is a crucial limitation of this measurement method regarding the planned application, see Section 2.3. Three image perspectives are required for out-of-plan 3D analyses [41]. The method is very sensitive to camera displacement [41]. This is considered as non-limiting, as surgical robotic systems with a highly accurate positioning are commercially available [61]. A stoichiometric pattern must be applied to the measurement surface [41]. Since sterile markers are commercially available [62], this is evaluated as solvable in principle. Image correlation is evaluated as potentially suitable.

#### 4.2.2. Non-Suitable Techniques

The lateral resolution of 0.1 mm of the *structured light method* is significantly lower than its vertical resolution [41]. Accordingly, this method is considered unsuitable for the planned application.

*Triangulation sensor* systems are likewise unsuitable due to their inability to simultaneously measure a 3D surface [41].

The synchronous acquisition of several measuring points is not possible when using a *scattered light sensor*. Additionally, the detector must be moved around the measuring object [41]. This contradicts the demanded small surgical access, see requirement No. 2, Table 3. Thus, this method is evaluated as unsuitable for the planned application.

*Laser tracking* might be somewhat suitable, but its application is complicated. A simultaneous lateral distance and temporally synchronous height measurement requires at least two separate measuring systems. Also, the implementation of mirrors on the measured object is required. Furthermore, the employed laser light might harm the subject’s tissue if not aimed at the mirrors. Due to these complexities, laser tracking seems marginally suitable at best.

*Autofocus optical systems* are incapable of measuring larger objects temporally synchronous [41]. This method is very sensitive to the motion of the measuring object [41]. In consequence, this method is considered unsuitable regarding the planned application due to vibrations caused, e.g., by the human heart.

*Both heterodyne and homodyne interferometry* require mirrors to be applied to the measuring object [41]. A miniaturization of those measuring mirrors is regarded as necessity for the planned application. Those methods are very sensitive to vibration and require laser radiation [41]. The laser radiation is potentially dangerous for tissue, if it does not hit the measuring mirrors. The method is evaluated to be possibly applicable for the planned application but technically very complex and, hence, impractical.

*Conoscopic holography* has a maximal lateral resolution of 0.025 mm [41]. Consequently, is does not fulfill the postulated minimal requirement of measurement accuracy.

Multiple wavelength interferometry suffers from several drawbacks: the optical detector needs to be moved around the object [41], temporally synchronous acquisition of a larger surface area is impossible [41], and the method is extremely sensitive to vibration-induced measurement errors [41]. These disadvantages render it unfit for the intended application.

*Maykoh sensors*, while demonstrating an acceptable vertical resolution, have an unsatisfactory lateral resolution of only 0.05 mm [41], thereby marking them as unsuitable.

*Holography*, highly sensitive to vibration and requiring the object to be rotated and shifted around the detector [41], is incompatible with the requirement for minimal surgical access as outlined in requirement No. 2 in Table 3. The need to track lateral displacement with 3D markers [41] further qualifies holography as unsuitable.

*Reflective photoelasticity* could be suitable, requiring a sterilizable and either degradable or reversible surface coating. The task of developing a surface coating with an extremely low Young’s modulus, while also maintaining suitable adhesive and optical properties, is deemed technically challenging. As a result, this technique is not viewed as a leading candidate for the intended application.

The *thermoelastic method* measures the warming of the measured object as a result of internal friction due to cyclic loading [41]. The implementation of cyclic loading within a surgical intervention is evaluated as problematic and technically complex. The time required is evaluated as unacceptable for the clinical routine. Additionally, the dissipation of heat from the measured object to the surrounding tissue is evaluated as problematic in regard to measurement accuracy.

### 4.3. Discussion

The measurement accuracy is evaluated as the most challenging technical aspect of an endoscopic system for a precise optical strain measurement in vivo for the postulated workflow describe in Section 2.1. A permissible measurement error based on the model assumption described in Section 2.2 is calculated as 1.90 × 10^−2 ^ mm to fulfill the minimal goal defined in Section 2.2. Ideally the measurement error should not exceed 9.75 × 10^−4^ mm. The predicted measurement accuracy is evaluated as technically challenging yet solvable. None of the screened medical endoscopic systems described in Section 3.1 and evaluated in Section 4.1 were identified as potentially suitable for the planned application. Three of the screened non-medical optical measuring systems (see Section 3.2 and Section 4.2) are potentially suitable for the described application. Namely, those methods are the confocal chromatic sensor, shearography, and image correlation. As seen in Section 3.3, confocal endoscopic systems are commercially available, but these systems are incapable for measuring a sufficient FoV.

Stress measurement based on reflective photoelasticity could provide the required information to describe the change in the strain state of a ligament surface, assuming linear elastic material behavior or exact knowledge of non-elastic material properties. 

Based on the calculated permissible measurement error, a minimum image resolution of 8.35 × 10^5^ pixels was determined in Section 2.3. Ideally, the resolution of an optical sensor should exceed 79 Megapixels, a challenge for endoscopic systems. However, this challenge is considered solvable, given the existence of CMOS sensors exceeding the anticipated required pixelation for the ideal case. Examples include the 122 Megapixel “120MXS CMOS” sensor by Canon Inc., Melville, NY, USA [63] and the 150 Megapixel “IMX411” sensor by Sony Semiconductor Solutions Corporation, Asahi-cho, Atsugi-shi, Kanagawa, Japan [64].

The objective of this study is to compare the technical opportunities for addressing the task outlined in Section 2.1. The technical screening was based on assumptions about measurement accuracy, related image resolution requirements, FoV, and the need for a non-destructive measurement method. The model assumption made in Section 2.2 is deemed suitable for most potential optical measurement setups, though not for all. This is considered the primary limitation of the methodology employed in this publication.

## 5. Conclusions

The non-destructive optical measurement of ligament strain via endoscopy, while technically challenging, appears feasible. Three methods emerged as promising candidates for accurate optical measurement of ligament strain alterations in the given scenario (refer to Section 2.1): confocal chromatic imaging, shearography, and image correlation. Confocal chromatic imaging necessitates 3D markers on the ligament surface for lateral displacement measurement, which is seen as a disadvantage compared to image correlation or shearography. Reflective photoelasticity for indirect strain measurement is deemed less desirable due to the requisite simplifications regarding material behavior or the need to ascertain additional tissue material properties.

Shearography, despite offering the highest potential for measurement accuracy and not requiring any tissue surface markings [41], involves the use of potentially harmful laser light [41] and a technically complex endoscopic system implementation, as assessed by the author.

Given the harmful laser light requirement for the first two methods [41], image correlation is considered the most promising approach. With the swift advancements in the resolution of CCD and CMOS sensors, there is potential for DIC to eventually meet the measurement accuracy of the ideal case. Considering the availability of precise robotic systems for endoscopic surgeries [61], this method stands out as the most promising technique currently available. Further investigations regarding the application of a stoichiometric pattern within MIS are required to realize a suitable measurement setup based on image correlation. 

## Figures and Tables

**Table 1:** Mandatory objectives for an interoperative measurement of ligament strain.

No.	Objectives
1	Non-destructive towards the ligaments tissue
2	No irreversible application of parts of the measurement setup on the ligaments tissue (such as markers, etc.)
3	Compatibility with minimal invasive surgery (MIS)
4	Minimal or no influence of the measurement setup on the measurement results
5	Compatibility with curvature of ligaments surface
6	No hindering of ligaments twisting
7	Measurement duration of 1–20 min
8	High measurement accuracy (see Section 2.2)

**Table 2:** Scientific methods for measuring ligament strain.

Refs.	Meas. Object	Application	Meas. Setup	Limitations for an Application in the Clinical Routine
[4,5,6,7]	Surface	Surgical suture	LMSG	- Risky in vivo due to the use of mercury; - Tissue damage from suturing the sensors; - No temperature compensation.
[8,9,10,11,12]	Surface	Barbs	HEST	- Tissue damage from barbs; - No temperature compensation; - Twisting of the ligament is hindered.
[11,12,13,14,15,16,17,18,19,20]	Surface	Barbs	DVRT	- Tissue damage due to barbs; - Twisting of the ligament is hindered.
[21,22,23,24,25]	Surface	Adhesive	Polymeric strain gauge with very low Young’s modulus	- Irreversible application through adhesive; - Manufacturing of the strain gauge not sterile so far.
[32,33]	Tissue	Adhesive	Fiber Bragg grating	- Irreversible application due to adhesive; - Tissue damage due to application.
[34]	Surface	Adhesive	Fiber Bragg grating	- Irreversible application due to adhesive.
[26,27]	Surface	Surface coating	Reflective photoelastic method	- Irreversible application of a non-biocompatible coating required; - Large access area required.
[31,32,33,34]	Surface	Needles/Adhesive for markers	DIC via CCD or CMOS sensors	- Tissue damage due to application of markers; - Large access area required; - Measurement accuracy insufficient for accurate determination of low strain rates and precise out-of-plane measurement.

**Table 4:** Case study for maximum permissible error of the measuring system Δx.

Case	$s_{A}^{*}$ in mm	ε	∆ε	*r*	∆x in mm
Ideal	10	0.05	0.001	5	9.75 × 10^−4^
Minimal	20	0.1	0.01	5	1.90 × 10^−2^

**Table 5:** Requirements for Image Resolution.

Case	Dimension of FoV		Pixel per Dimension	Total Number of Pixel
		In mm		
Ideal	Length	15	1.54 × 10^4^	7.89 × 10^7^
	Width	5	5.17 × 10^3^	
Minimal	Length	30	1.58 × 10^3^	8.35 × 10^5^
	Width	10	5.27 × 10^2^	

**Table 6:** Keywords of PubMed recherche.

Keyword	Keyword
3D endoscope development	Endoscope holography
Endoscope structured light 3D	Endoscope shearography
Endoscope strain measurement	Endoscope Speckle pattern shearing interferometry
Endoscope diffractive optical element	Endoscope confocal

**Table 7:** Screening result for potentially applicable medical endoscopic systems.

Refs.	Technique	Max. Error of Measuring System in mm	FoV in mm × mm
[42]	Laser pattern	-	-
[43]	Active stereo	0.3–0.4	-
[44]	Active stereo	-	-
[45]	Multi view stereo, structure from motion	0.2–0.3	-
[46]	Photometric stereo	0.5	-
[47]	Weighted orthogonal-symmetric local binary pattern (WOS-LBP), multi view stereo	0.03	-
[48]	Structured light projection	0.3	-
[49]	Structured light projection	0.25	-
[50]	Holography	0.0022	0.390 × 0.244
[51,52]	Grid pattern projector, active stereo	-	-
[53]	Shapes from shading	0.3	-
[54]	Structured light projection	0.092	30 × 30
[55]	Structured light projection	-	-
[56]	Structured light projection	0.15	-
[57]	Shapes from shading	1.45	-
[58]	Confocal laser	0.0035	-
[59,60]	Confocal laser	0.0007	0.475 × 0.475

**Table 8:** Screening results for non-medical optical measurement systems.

Method	Measurement Accuracy Criterion		FoV Criterion (Lateral)	
	Ideal	Minimal	Minimal	Ideal
	Δx<0.001 mm	Δx<0.02 mm	$>3\times 10^{-4}\;m^{2}$	$>7.5\times 10^{-5}\;m^{2}$
Structured light projection	No	Yes	Yes	Yes
Triangulation sensor	Yes	Yes	Yes	Yes
White light interferometry	Yes	Yes	No	No
Confocal microscopy	Yes	Yes	No	No
Confocal chromatic sensors	Yes	Yes	No	Yes
Scattered light sensor	Yes	Yes	No	Yes
Laser tracker	No	Yes	Yes	Yes
Autofocus optical system	Yes	Yes	Yes	Yes
Heterodyne and homodyne interferometry	Yes	Yes	No	Yes
Conoscopic holography	Yes	Yes	Yes	Yes
Ellipsometry	Yes	Yes	No	No
Multiple-Wavelength Interferometry	Yes	Yes	Yes	Yes
Maykoh sensor	Yes	Yes	Yes	Yes
Shadow casting method	Yes	Yes	No	No
Holography	Yes	Yes	Yes	Yes
Shearography	Yes	Yes	Yes	Yes
Image correlation	No	Yes	Yes	Yes

**Figure A1:**
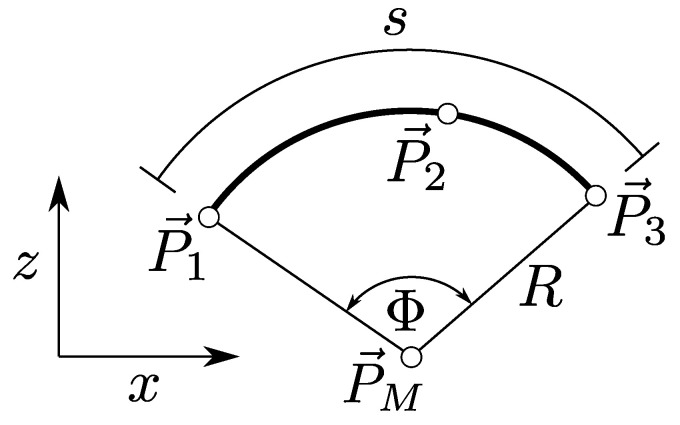
Simplified model of the ligament surface.

**Figure A2:**
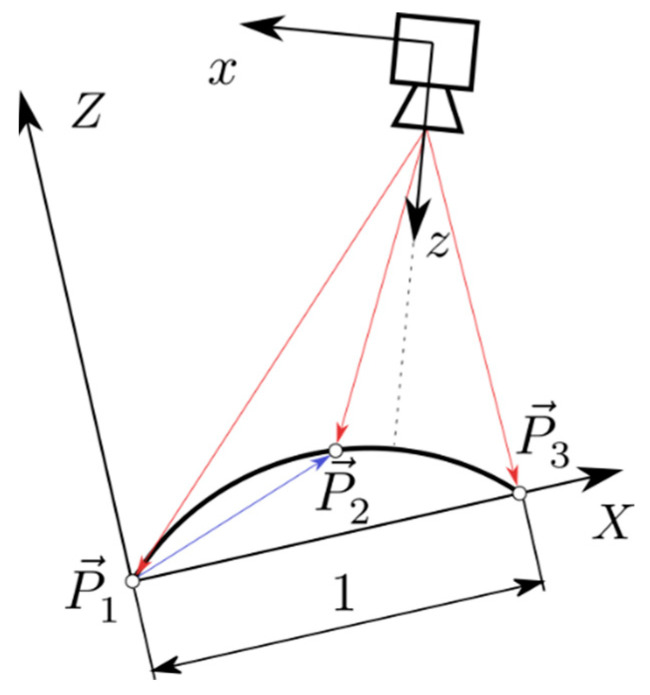
Adjusted coordinate system.

**Figure A3:**
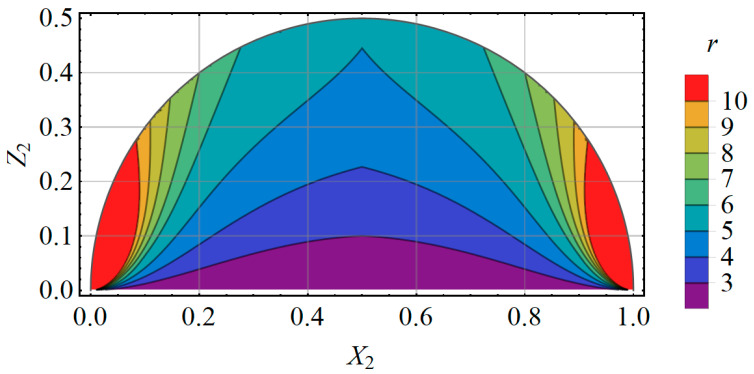
Error ratio r in the considered range of validity.

## Data Availability

No new data were created or analyzed in this study. Data sharing is not applicable to this article.

## Appendix A

### Appendix A.1. Consideration of the Extreme Case

To identify the most critical influence of the measurement error of the length ∆s, two different extreme cases are considered. Either ∆s is added to state A and subtracted from B or vice versa:

(A1) $$\varepsilon =\frac{\left(s_{B}^{*}\pm ∆s\right)-(s_{A}^{*}\mp ∆s)}{s_{A}^{*}\mp ∆s}\;.$$

Equation (1) is inserted into Equation (A1):

(A2) $$\Delta \varepsilon =\left|\varepsilon -\varepsilon^{*}\right|=\left|\frac{\left(s_{B}^{*}\pm ∆s\right)-\left(s_{A}^{*}\mp ∆s\right)}{s_{A}^{*}\mp ∆s}-\frac{s_{B}^{*}-s_{A}^{*}}{s_{A}^{*}}\right|=\frac{∆s(s_{A}^{*}+s_{B}^{*})}{s_{A}^{*}(s_{A}^{*}\mp ∆s)}.$$

Based on Equation (A2), it becomes clear that the more critical case arises in each case with the upper operation sign. Accordingly, only this one will be considered in the following. Utilizing Equation (2), the strain deviation can be expressed as follows:

(A3) $$∆\varepsilon =\frac{∆s(2+\varepsilon )}{s_{A}^{*}-∆s}.$$

### Appendix A.2. Mathematical Description of the Circular Segment via Three Spatial Measurement Points

Within this section a general description of how to determine the length $s_{A}$ or $s_{B}$ of a ligament segment with the aid of three measuring points for one of the two states A and B is described. The indices for the separation of state A and B are not used in the following section. To uniquely determine the length of a circular segment s, three points ${\vec{P}}_{i}$ on the ligament surface must be known:

(A4) $$\;\;{\vec{P}}_{i}=\left(\begin{array}{c} x_{i}\\ z_{i} \end{array}\right)\;,\;\;i=1,2,3.$$

The circle equation belonging to the circle segment is as follows:

(A5) $$\;R^{2}={\left(x-x_{M}\right)}^{2}+{\left(z-z_{M}\right)}^{2}.$$

Here R is the radius of the circle; $\;x_{M}$ and $z_{M}$ are the coordinates of the center $\;{\vec{P}}_{M}$ in the selected coordinate system. Using the points from Equation (A4), a nonlinear system of equations is obtained to determine these quantities. The solution of the system is shown below:

(A6) $$R=\frac{1}{2}\sqrt{\frac{\left((x_{1}-x_{2}{)}^{2}+{\left(z_{1}-z_{2}\right)}^{2}\right)\left({\left(x_{1}-x_{3}\right)}^{2}+{\left(z_{1}-z_{3}\right)}^{2})((x_{2}-x_{3}{)}^{2}+{\left(x_{2}-z_{3}\right)}^{2}\right)}{{\left(x_{1}\left(z_{3}-z_{2}\right)+x_{2}\left(z_{1}-z_{3}\right)+x_{3}\left(z_{2}-z_{1}\right)\right)}^{2}}}\;,$$

(A7) $$x_{M}=\frac{(z_{2}-z_{3})\left(x_{1}^{2}+\left(z_{1}-z_{2}\right)\left(z_{1}-z_{3}\right)\right)+x_{2\;}^{2}\left(z_{3}-z_{1}\right)+x_{3\;}^{2}\left(z_{1}-z_{2}\right)}{2(x_{1}\left(z_{2}-z_{3}\right)+x_{2}\left(z_{3}-z_{1}\right)+x_{3}\left(z_{1}-z_{2}\right))},$$

(A8) $$z_{M}=\frac{x_{1}^{2}\left(x_{3}-x_{2}\right)+x_{1}\left(x_{2}^{2}-x_{3}^{2}+z_{2}^{2}-z_{3}^{2}\right)-x_{2}^{2}x_{3}+x_{2}\left(x_{3}^{2}-z_{1}^{2}+z_{3}^{2}\right)+x_{3}\left(z_{1}-z_{2}\right)\left(z_{1}+z_{2}\right)}{2(x_{1}\left(z_{2}-z_{3}\right)+x_{2}\left(z_{3}-z_{1}\right)+x_{3}\left(z_{1}-z_{2}\right))}.$$

Thus, the associated circle equation is unambiguously determined. Note that, sensibly, only the positive value of *R* is given. Between the outer points of the segment (here, ${\vec{P}}_{1}$ and ${\vec{P}}_{3}$) spans the angle Φ, as seen in Figure A1. It can be calculated as

(A9) $$\Phi =\cos^{-1}\left(\frac{\left({\vec{P}}_{1}-{\vec{P}}_{M})\cdot ({\vec{P}}_{3}-{\vec{P}}_{M}\right)}{\left|{\vec{P}}_{1}-{\vec{P}}_{M}\right|\left|{\vec{P}}_{3}-{\vec{P}}_{M}\right|}\right),$$

where “·” represents the scalar product. Finally, the quested length *s* of the circle segment is found as seen in Equation (A10):

(A10) s=RΦ.

### Appendix A.3. Mathematical Description of the Measuring Errors of the Arc Length s

This section describes how an incorrect determination of the point coordinates affects the error in the length of a circle segment ∆s. The resulting measurement error of the arc length s can be expressed in terms of the measurement error in the spatial coordinates of the points $\;∆\vec{P_{i}}$ = (Δ*x_i_*, Δ*z_i_*) for *i* = 1, 2, and 3. According to [65], the ordinary sum as seen in Equation (A11) is utilized to determine the maximum measurement error ∆s:

(A11) $$∆s=\sum_{i=1}^{3}\left(\left|\frac{\partial s}{\partial x_{i}}\right|∆x_{i}+\left|\frac{\partial s}{\partial z_{i}}\right|∆z_{i}\right)\;.$$

The assumption is made that the measurement errors are all the same magnitude in the spatial coordinates (Assumption IV):

(A12) $$∆P_{i}=∆x_{i}=∆z_{i}\;.$$

According to assumption IV an error ratio r can be defined as follows:

(A13) $$r≔\frac{∆s}{∆x}=\sum_{i=1}^{3}\left(\left|\frac{\partial s}{\partial x_{i}}\right|+\left|\frac{\partial s}{\partial z_{i}}\right|\right).$$

As the ratio ∆s*/*∆x defines a relative relationship, it does not change by repositioning (rotation, translation) or scaling the coordinate system. On the other hand, there is still the dependence on the position variables $x_{i}$ and $z_{i}$. From this it can be concluded that r can also be parameterized other than by six independent variables.

### Appendix A.4. Choice of a Suitable Coordinate System

All the previous relationships apply to an arbitrary Cartesian coordinate system. Now, the error ratio is to be parameterized as suitable. From now on we distinguish between a camera coordinate system (x,z) and a dimensionless, problem-specific coordinate system (X,Z); see Figure A2. The latter is chosen in such a way that its origin lies in the point ${\vec{P}}_{1}$. The x-axis shall point to ${\vec{P}}_{3}$. Furthermore, the X-coordinate of ${\vec{P}}_{3}$ shall take the value 1.

In the adjusted coordinate system, the three points are as follows:

(A14) $${\vec{P}}_{1}=\left(\begin{array}{c} 0\\ 0 \end{array}\right)\;,{\vec{P}}_{2}=\left(\begin{array}{c} X_{2}\\ Z_{2} \end{array}\right)\;,\;{\vec{P}}_{3}=\left(\begin{array}{c} 1\\ 0 \end{array}\right)\;.$$

This approach allows a graphical representation of r as a function of the free parameters $X_{2}$ and $Z_{2}$:

(A15) $$r={\left.\sum_{i=1}^{3}\left(\left|\frac{\partial s}{\partial X_{i}}\right|+\left|\frac{\partial s}{\partial Z_{i}}\right|\right)\right|}_{{\vec{P}}_{1},{\vec{P}}_{2},{\vec{P}}_{3}}\;.$$

In Figure A3, the error ratio r is shown over the maximum detectable range of the camera from $0<X_{2}<1$ and $Z_{2}<\sqrt{X_{2}-X_{2}^{2}}$.

As mentioned before, neither a change in position nor a scaling of the coordinate system changes the ratio of ∆s/∆x. Consequently, the relation from Equation (3) is valid especially for the cameras Cartesian coordinate system.

### Appendix A.5. A Prediction for the Measuring Error of the Technical System

Based on the assumptions made so far, an estimate for the maximum permissible error of the measuring system Δx is obtained, starting from a given minimum requirement for the strain deviation ∆ε. The error ratio r is used to substitute ∆s into Equation (3) and to solve for the quantity we are looking for:

(A16) $$∆x=\frac{s_{A}^{*}∆\varepsilon }{\left(2+∆\varepsilon +\varepsilon \right)r}\;\;.$$

Regarding the error ratio r, the following assumptions are made. The *X*-value of measurement point 2 is within the range of 0.4–0.6 within the adjusted coordinate system (Assumption V) as this is a realizable accuracy to meet the middle position of the measuring area, even if the stoichiometric pattern is applied manually. A maximal curvature of the ligament should not exceed a ratio of *Z*_2_ of 0.3 within the adjusted coordinate system. Accordingly, the error ratio r should not exceed a magnitude of 5, see Figure A3.
